# Delineating profit gaps in rice and wheat production: evidence from India’s economically marginalized region

**DOI:** 10.1186/s40100-025-00442-x

**Published:** 2025-12-01

**Authors:** Anurag Ajay, Maxwell Mkondiwa, Anton Urfels, Daniel Müller

**Affiliations:** 1 Leibniz Institute of Agricultural Development in Transition Economies, Halle, Germany; 2 Humboldt-Universität zu Berlin, Berlin, Germany; 3 International Maize and Wheat Improvement Center, New Delhi, India; 4 International Rice Research Institute, Los Baños, Philippines; 5 Wageningen University and Research, Wageningen, The Netherlands; 6 Integrative Research Institute on Transformations of Human-Environment Systems (IRI THESys), Humboldt-Universität zu Berlin, Berlin, Germany

**Keywords:** Bihar, Eastern India, Profit efficiency, Sources of inefficiency, Stochastic frontier

## Abstract

Most farms in the Global South are small family farms and supporting these in generating ideal profits is critical for reducing poverty. We study the state of Bihar, the poorest in India where approximately 100 million people source their food and income from farms smaller than two hectares. Our research assesses the profit efficiencies achieved by smallholders in rice and wheat production and examine the variation in profit efficiencies across different farm sizes and geographical regions of the state. Applying single-step stochastic profit frontier analysis with data from 4016 randomly selected farmers, we estimate profit potential and identify sources of profit inefficiency. Spatial mapping with profit efficiency scores helps visualize differences and pattern within the state. The findings reveal that high planting cost in wheat and high harvesting costs in rice and wheat reduce profit efficiency. Additionally, excessive rainfall in August and higher temperature in November impede profit efficiency of rice and wheat, respectively. The biggest source of inefficiency in rice and wheat production is costly diesel-driven irrigation system. Our estimates suggest that closing profit efficiency gaps can increase smallholder profits by 38% in rice and 35% in wheat. Identifying strategies to close profit gaps in smallholder settings and agriculture-led economies is crucial for informing interventions that alleviate rural poverty.

**Graphical abstract:**

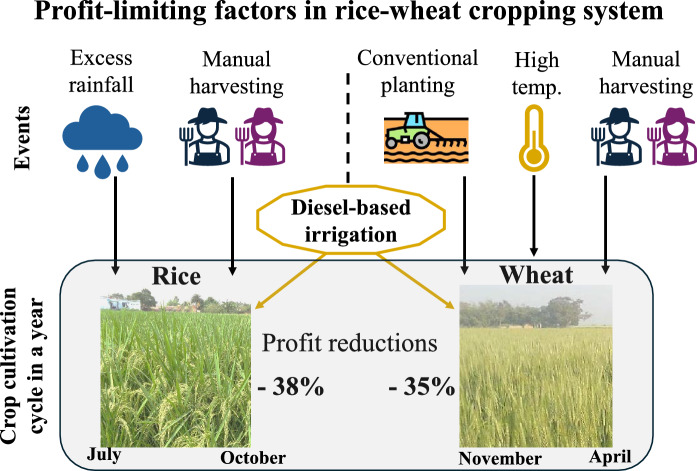

## Introduction

A large proportion of farming communities in the Global South depend on landholdings smaller than two hectares (ha), commonly referred to as small farms. These farms serve as the primary source of food and income for millions of households. Globally, 84% of 608 million farms are small farms, contributing 35% of the world’s food supply (Lowder et al. 2021). Smallholders operate around 40% of the agricultural land in South Asia, East Asia, Sub-Saharan Africa, and Latin America (Lowder et al. 2016). Their role in agricultural development in these regions is crucial due to their sizable presence. However, they encounter numerous challenges and risks that constrain their farm outputs and eventually household income (Mujuru et al. 2022; Touch et al. 2024). Examining what can sustainably enhance their performance remains essential for supporting agricultural development, especially in low- and middle-income countries where smallholders are prevalent.

In India, the significance of smallholder farming is even more pronounced. Nearly two-thirds of country’s 1.4 billion people depend on agriculture for their livelihoods. Of the 146 million farms, 87% are small farms. The average landholding of Indian farmers is 1.08 ha and 69% of them operate on less than one hectare (Ministry of Agriculture 2019). Such fragmented holdings often fail to generate enough output that can meet household consumption needs (Manjunatha et al. 2013). Limited landholdings restrict production potential by limiting farmers’ ability to invest in technology and farms in general. This situation contributes to India’s persistently high levels of poverty among farming communities (Roy and Divyanshi 2019). In this context, scrutinizing income enhancing opportunities from small farms is therefore pertinent for informing polices aimed at alleviating rural poverty.

The income of Indian farmers remains critically low due to low farm profitability. In 2016, the average monthly income of Indian farmers was US$105 with large interstate disparities (Gulati et al. 2021). The states in eastern India such as Bihar, Jharkhand, Odisha, and West Bengal are hotspots of poverty because farmers’ average monthly income is less than US$90. Moreover, the rising share of off-farm income in total household income highlights the insufficiency and instability of farm-based revenues and profits (Harkness et al. 2021). Recognizing the centrality of farmers’ income, the Government of India took several initiatives, including the nationwide program on ‘doubling farmers’ income’ (Bihari et al. 2019) and the ‘PM-KISAN’ scheme to strengthen farmers’ working capital (Kavitha et al. 2020). However, region-specific tailored interventions are needed to address structural constraints.

Profit from crop production is a key indicator of farm success, shaped by production planning, management of resources, natural conditions, and structural arrangements (Li and Li 2018). In a farming household, apart from meeting consumption needs, better profit enables reinvestments in farm production and pursues off-farm opportunities (Birthal et al. 2014). Despite its importance in farmers’ welfare, evidence on profit efficiency remains sparse due to lack of field-level data on crop production, costs, and prices at a reasonable scale particularly in smallholder’s environment.

Profit efficiency evaluates the overall economic success of the farm. It reflects the ability of a farm to optimize input use for higher economic returns. Measuring profit efficiency helps in quantification of untapped profit potential and reveal inefficiency sources (Kumbhakar 2001). Profit efficiency of a farm is governed mainly by input costs, amount of produce, and output prices. However, inefficiency might be caused by external factors such as soil fertility, characteristics of households, and farm characteristics (Adnan et al. 2021; Ngeno 2024; Muleta and Mebratu 2024). Identifying these determinants is crucial for developing context-specific policies that enhance profitability of farms.

Regional variations in natural conditions, demographics, and farming methods can significantly influence farm outcomes. Beyond the management of allocative resources, profit efficiency is also shaped by these intrinsic regional characteristics. It means that identical crop management practices may result in different outputs and profit efficiencies across locations. Approaches addressing geographical differences in profit efficiency would help in identifying and prioritizing pockets that require greater attention (Akite et al. 2022). Such insights can guide targeted interventions, ensuring that policies and investments are directed where they can most effectively enhance farmers’ profitability and rural livelihoods.

Our study examines profit efficiency in the production of rice and wheat which are cultivated in rotation throughout an agricultural calendar year in India. We apply the stochastic frontier approach (SFA) to estimate the attainable profit and reveal the sources of inefficiency (Lovell 1993). We use cross-sectional survey data from 4016 farmers collected in the state of Bihar in India. We quantify how much additional profit is potentially attainable at the current level of input use, its determinants and sources of inefficiency. Furthermore, we provide insights on how profit efficiency differs across farm sizes and geographical regions. The overall aim of our study is to generate evidence that can inform policies for supporting farm profitability and contribute to reducing poverty in Bihar. Our research answers following three questions:

A. What profit efficiencies do smallholders achieve in production of rice and wheat in a rotational system over one agricultural calendar year?
B. What are the key determinants of profit efficiencies and sources of inefficiency in the rice–wheat rotational system dominated by smallholders in Bihar?
C. How do the profit efficiencies of rice and wheat production vary across farm sizes and geographical regions within Bihar, India?

## Rice–wheat cropping system in eastern India

The rice–wheat rotational system is the most prevalent cropping system along the Indo-Gangetic Plains that stretches up to mid-hills of the Himalayas (Kataki et al. 2001). This cropping system spans 15.8 million ha across Bangladesh, India, Nepal, and Pakistan, with India accounting for 78% of the area (Nadeem and Farooq 2019). Two-thirds of India’s food grain is produced by this system, highlighting its importance for India’s food self-sufficiency (Mahajan and Gupta 2009). Particularly in Bihar where smallholders constitute 97% of farming households, it is the primary source of food, income, and employment.

The rice–wheat cropping system refers to cultivation of rice followed immediately by wheat on the same field in a full calendar year. In eastern India, the cycle begins with setting up the rice nursery in June on a sub-plot, preferably at a site where it can be frequently watered. When the monsoon peaks in July, the nursery is carefully uprooted and manually transplanted on the main field. Other main in-season activities include irrigation (depending on rainfall frequency), fertilization (typically three times), and weeding (usually twice) throughout the rice-growing season. Single spray of weedicide is common, but the application of insecticide and fungicide are incidence-based. As the monsoon recedes completely in October, the rice crop reaches maturity, and the harvesting process begins.

Preparation of wheat planting kicks off in November shortly after the rice harvest. The field is generally plowed several times using a tractor-mounted cultivator and wheat seeds along with fertilizers are evenly broadcasted in the field. Although a mechanical seed-cum-fertilizer drill is available for wheat planting (Gupta et al. 2019). Irrigation is the most critical in-season operation because of the dry winter. Most farmers apply irrigation three times throughout the wheat growing period, despite the five recommended irrigations. With the arrival of summer in March, the wheat crop enters its final development stage and its harvesting starts at the end of April. The field remains fallow for the remaining two months until the next rice season commences. In this production system, farmers’ year-round profit is determined by the efficiency with which they produce these two crops.

## Methodology

### Conceptual framework of profit efficiency

Gross profit is calculated as total revenue minus all variable costs in crop production. Net profit is derived from gross profit by subtracting depreciation on farm machinery and infrastructure, which constitute fixed costs. We calculated the total revenue from fields by multiplying grain output with its selling price, typically called the farm gate price (FGP). Production cost, in the system with limited ownership of large machinery, primarily consists of labor and material inputs. Due to limited capital and poor access to credit, smallholders rarely own large farm machinery or invest in farm infrastructures; hence, fixed costs are typically minimal. As a result, gross and net profits are nearly the same (referred to as profit in our study). In cases where machinery was used, we considered rental costs that accounted for depreciation.

**Fig. 1:**
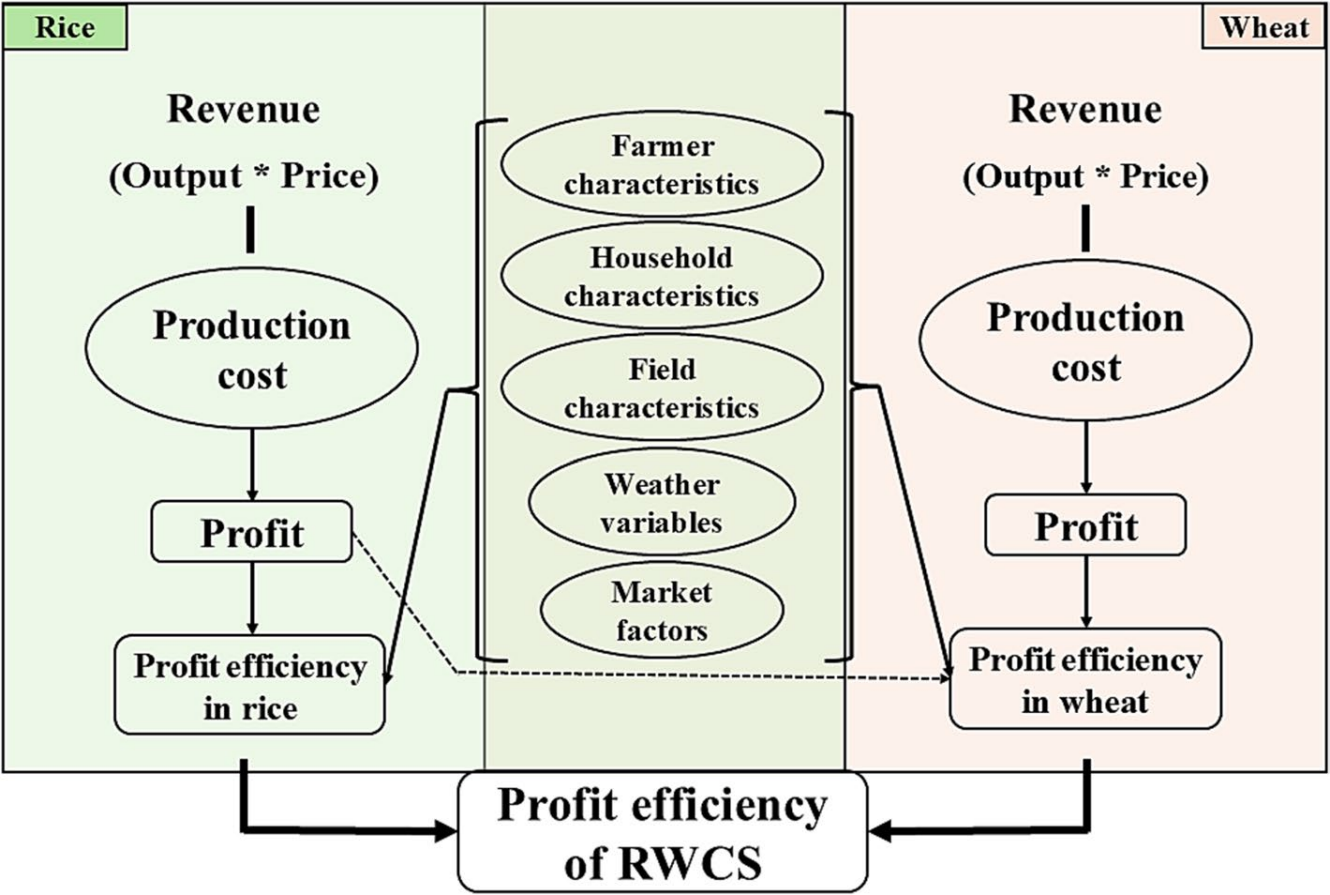
Conceptual framework defining profit efficiency in the rice–wheat cropping system (RWCS) and its key constituents

Profit efficiency, the ratio of actual and potential profits, is a valuable measure of field performance, reflecting how efficiently inputs translate into generating profit. Even if two fields use the same amount of input, their profit efficiencies may differ due to various factors such as the methods of input application, timings of farm operations, local farm conditions, weather, and selling prices. Profits from the rice harvest govern the option space of farming decisions for the wheat season, thereby modulating the potential profits from wheat production. Together, the two crops, determine yearly profit from the rice–wheat cropping system (Fig. 1).

### Data

#### Study area

We studied the state of Bihar located in the eastern part of India. With a geographical coverage of 94,000 km^2^, 60% of its land is arable. It is India’s third most populous state with 104 million people, projected to have reached 130 million by 2024 (Population Census 2011). Almost 90% of the population resides in rural areas, with agriculture and allied activities as the primary source of livelihood. Agriculture contributes 26% to Bihar’s gross domestic product, with smallholders dominating the agricultural economy. These farmers, who hold less than 2 ha, make up 97% of the farming population and cultivate 76% of the farmland (Ganguli and Sinha 2014). Farmers are categorized by farm size into marginal, small, medium, and large. The average income of farmers in Bihar is US$1.5 per day, and half of the state’s population is characterized as multidimensionally poor (UNDP 2023). Agricultural development is essential for both the welfare of the people and the economic progress of Bihar.

**Fig. 2:**
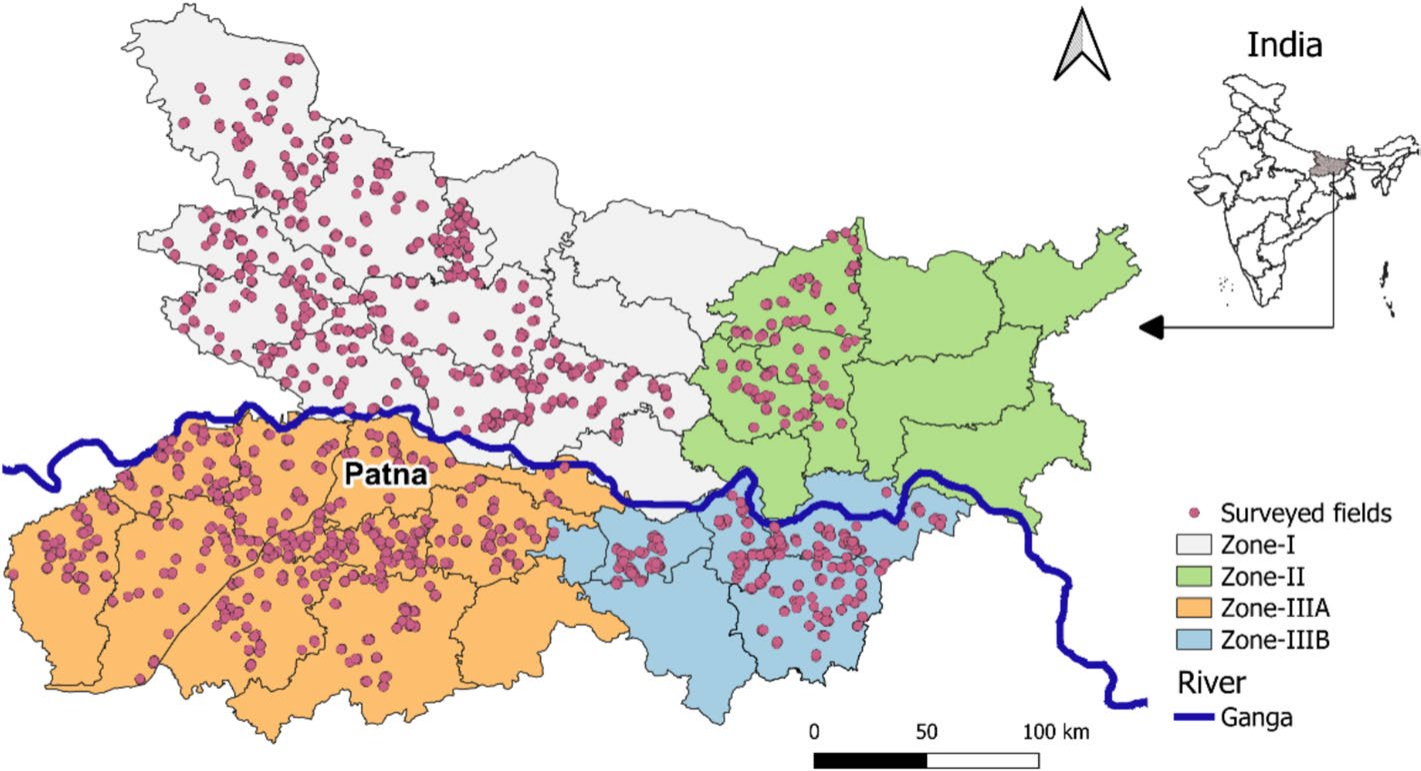
Study area map highlighting the state of Bihar, India with district boundaries; dots represent locations of surveyed rice–wheat fields

Bihar is a flat plain divided into two equal parts (North and South Bihar) by the river Ganga flowing west to east (Fig. 2). Based on agroclimatology, it has four zones: the north alluvial plain (Zone-I), northeast alluvial plain (Zone-II), southwest alluvial plain (Zone-IIIA), and southeast alluvial plain (Zone-IIIB). There are 38 geographically distinct administrative units referred to as districts. Our study covered 26 of these districts.

#### Sampling technique and survey

Data collection employed a two-stage cluster sampling technique to randomly select villages and farmers (Makela et al. 2018). The survey, known as the Landscape Diagnostic Survey, was part of CSISA project (www.csisa.org) in India. Villages were selected from the sampling frame constructed from the Indian village census 2011 using probability proportionate to size method (Latpate et al. 2021). Farmers were then drawn randomly from the electoral rolls of the chosen villages. Accordingly, in the first stage, 30 villages were selected for each of the 26 districts, totaling 780 villages. Subsequently, six farmers were identified from each of these villages.

We collected cross-sectional survey data from 4016 farmers in 2018 through personal interviews conducted by trained enumerators. They recorded farmers’ responses using a structured digital questionnaire designed on Open Data Kit platform (Brunette et al. 2013). Data were collected for the largest field where farmers cultivated both rice and wheat in rotation. The survey comprised various modules covering questions on planting, fertilizer management, irrigation, weed control, harvesting/threshing, production, and sales. Separate sets of questions were asked for rice and wheat though they followed a similar logical flow. Production cost data, reflecting the survey period, was gathered in 2023 through district-level expert consultations assuming no intra-district variability. The cost data that included all material inputs, labor, and machine rentals, was integrated into the farmers’ survey dataset for analysis.

#### Weather and soil data

We accessed field-level data on rainfall, temperature, and soil from secondary sources using the field-specific geo-coordinates recorded during the survey. We extracted rainfall data from the Climate Hazards Group Infrared Precipitation with Stations (CHIRPS) dataset for the rice-growing period (Funk et al. 2015). CHIRPS uses infrared Cold Cloud Duration and interpolation techniques, which are assessed reliable and valid for India (Prakash 2019). Similarly, location-specific temperature data was obtained from climate data of the Copernicus Climate Change Service (C3S) for the wheat growing period. The ERA5 product of C3S relies on an integrated forecasting system that significantly enhances horizontal resolution and hourly output (Hersbach et al. 2020). The average monthly rainfall and temperature of each field were converted into indices. We constructed wetness and heat indices by dividing each field-specific actual monthly reading of rainfall and temperature with the long-term average derived from the past 30 years (Zhang et al. 2011). Data on the status of field’s soil physical and chemical properties came from the digital soil map of Bihar (ISRIC 2024). Weather and soil datasets were clubbed together using geo-coordinates of the fields and eventually integrated with the survey data for analysis.

#### Data analysis

We applied a single-step stochastic frontier model developed for cross-sectional data using the statistical software Stata (Belotti et al. 2013). We tabulated descriptive statistics of the target, independent, and control variables of rice and wheat. The target variable, profit, was calculated as revenue minus costs, where revenue was obtained by multiplying farmer-level selling prices with respective crop yields. We separately measured the profit efficiency of rice and wheat fields, estimated the effect size of parameters, and identified sources of inefficiency. We then map the efficiency scores into a combined profitability map of rice and wheat at the district level.

### Profit efficiency and frontier

Profit efficiency refers to the ability of managing resources and producing outputs with higher economic value. In crop production, it attributes to farmers’ managerial capabilities in optimizing input use and applying practices intended toward securing maximum output. Profit frontier, on other hand, is a given set of inputs, resources, and markets that help in achieving attainable profits. Profit frontier is a measure of the distance between current and attainable profit (Kumbhakar et al. 2015).

Conventionally, the efficiency is categorized into technical and allocative efficiency. Technical (or production) efficiency reflects the ability to attain the highest production level with a given set of inputs (Russell and Young 1983). Allocative (or cost) efficiency captures the input use that minimizes production costs (Kalirajan 1985). Profit efficiency integrates both technical and allocative components. So, attaining a high profit efficiency requires both cost minimization and revenue maximization aspects. Accordingly, the measurement of profit efficiency considers errors on both input and output sides. It is thus a more appropriate indicator for evaluating economic performance of farm. The most widely used techniques to measure profit efficiency are stochastic frontier (parametric) and data envelopment (non-parametric) analyses. The latter being less applicable in typical economic objectives including profit maximization (Berger and Humphrey 1997). In line with our research objectives, we employed a stochastic profit frontier to estimate the maximum potential profit that farmers could achieve, given input prices, management practices, and soil and environmental conditions (Kumbhakar 1994).

#### Profit efficiency with stochastic frontier model

The stochastic frontier model assumes that the maximum profit can only be achieved for a given set of inputs without inefficiency. The model estimates frontier, sometimes referred to as boundary, using maximum likelihood estimation to infer frontier parameters. Departures from this boundary are attributed to error term which consists of two components—one is random error associated with statistical noise (measurement error) and the other represents systematic effects caused by inefficiency factors (Coelli et al. 2005). The foundational formula of the stochastic frontier model is given in Eq. (1).

1 $$p_{i} = \alpha + \beta^{\prime}x_{i} + V_{i} - U_{i}$$

where $p$ is the profit from $i$th field, $\alpha$ is the constant term (intercept) in the model, ${\beta }{\prime}$ vector of coefficients associated with the input variables, ${x}_{i}$ is a vector of inputs for $i$th field, ${V}_{i} and {U}_{i}$ are the error components. ${V}_{i}$ is statistical noise, whereas ${U}_{i}$ is a non-negative term accounting for inefficiency. They together define the distance by which the observed field fails to reach the frontier. ${\beta }{\prime}{x}_{i}+{V}_{i}$ constitute the stochastic frontier.

As the stochastic profit frontier and inefficiencies are estimated simultaneously, the composite error term is expressed in variance parameters as described in Eq. (2).

2 $$\gamma = \frac{{\sigma_{u}^{2} }}{{\sigma^{2} }} \,{\text{and}}\, \sigma^{2} = \sigma_{v}^{2} + \sigma_{u}^{2} , \,{\text{where}}\, 0 \le \gamma \ge 1$$

The value of $\gamma$ closer to 1 implies that the variation is attributed more to inefficiency, whereas its proximity to 0 symbolizes more variation is due to statistical noise (Skolrud 2016).

#### Empirical single-step stochastic profit frontier model

We applied the stochastic profit frontier with a Cobb–Douglas functional form because the exponents directly represent the input–output elasticity and constant substitution elasticity is reasonable (Douglas 1928). Compared to another substitutes such as semi–log and no–log, the log–log or trans–log functional form is a better fit in profit frontiers according to the generalized likelihood ratio test (Kaka et al. 2016). It is not restricted to the assumptions about the curvature of the frontier and represents the real-world production environment involving single output and multiple inputs (Appelbaum 1979). However, we compared the results of trans–log form with semi–log and no–log forms to cross-validate the results. We calculated profit efficiencies using the Jondrow, Lovell, Materov, and Schmidt (JLMS) method (Jondrow et al. 1982).

Variables contributing directly to the profits were included in the deterministic component, while structural and farm-specific characteristics influencing performance were accounted in the inefficiency term (Kolawole 2006; Greene 2008). Accordingly, our empirical model included all cost variables (field preparation, planting, seed, fertilizers, irrigation, weeding, and harvesting) and field-specific natural parameters (rainfall in rice, temperature in wheat, and soil). Exogeneous variables (field size, field ownership type, irrigation pump type, seed type, distance of input market, education of farmer, family size, and caste of farmer.) were supplied into the inefficiency function specification. This model allowed the estimation of field-specific efficiency scores and the sources explaining inefficiency among farmers in a single-step estimation procedure. The model for wheat consisted of profit earned from previous rice, while the inefficiency component remained the same as of rice. The empirical profit frontier models of rice and wheat are furnished in Appendix 1.

#### Unobserved heterogeneity and spatial dependence

To account for unobserved heterogeneity in the model, we considered potential correlation in unobserved factors across villages. Specifically, we employed cluster-robust standard errors, allowing for intra-village correlation while maintaining consistency of inference (Abadie et al. 2023). This adjustment acknowledges that unobserved village-level characteristics (microclimatic conditions or local agricultural information system) may influence farm-level outcomes (Mittal et al. 2018).

We examined the presence of spatial dependence on efficiency scores using Moran’s I test (Chen 2013). This test (20-nearest neighbors’ spatial weight matrix) evaluated whether efficiency estimates exhibit systematic spatial clustering across neighboring fields. The results did not provide strong evidence of spatial autocorrelation, suggesting that efficiency scores were largely independent across space and that village-level unobserved factors were already sufficiently captured by our model specification.

## Results

### Descriptive statistics

Demographically, the farmers in the sample owned an average field size of 0.21 ha, with an overall farm size of 1.14 ha. The majority (88%) were smallholders, including 62% classified as marginal farmers (farm size below 1 ha). A small fraction (14%) of farmers had rented-in land for cultivation, indicating that they were either landless or had not enough farmland for sustenance. From a social perspective, the majority (58%) belonged to the Other Backward Class, a group that represents middle tier in the Indian caste system. More than one quarter of the respondents had no formal education, while another 31% had studied only up to class five (primary education level). On average, there were eight members per family, which is higher than the Indian average of five.

The average yield of rice and wheat were 4.0 and 2.9 ton per hectare (t·ha^−1^), respectively, which generated corresponding gross revenues of US$770 and US$560 per ha. The average production cost of rice was US$185 per ha more and the selling price US$25 per ton lesser than that of wheat. The largest cost factor in rice production was associated with harvesting, followed by irrigation and planting. In the case of wheat, irrigation cost was comparatively lower but planting and harvesting costs jointly accounted for 59% of the total cost of wheat production. We observed that majority of farmers use diesel-powered irrigation pumps in both the crops. Descriptive statistics of rice and for wheat are available in Appendix 2.

### Profit efficiencies of rice–wheat fields

**Fig. 3:**
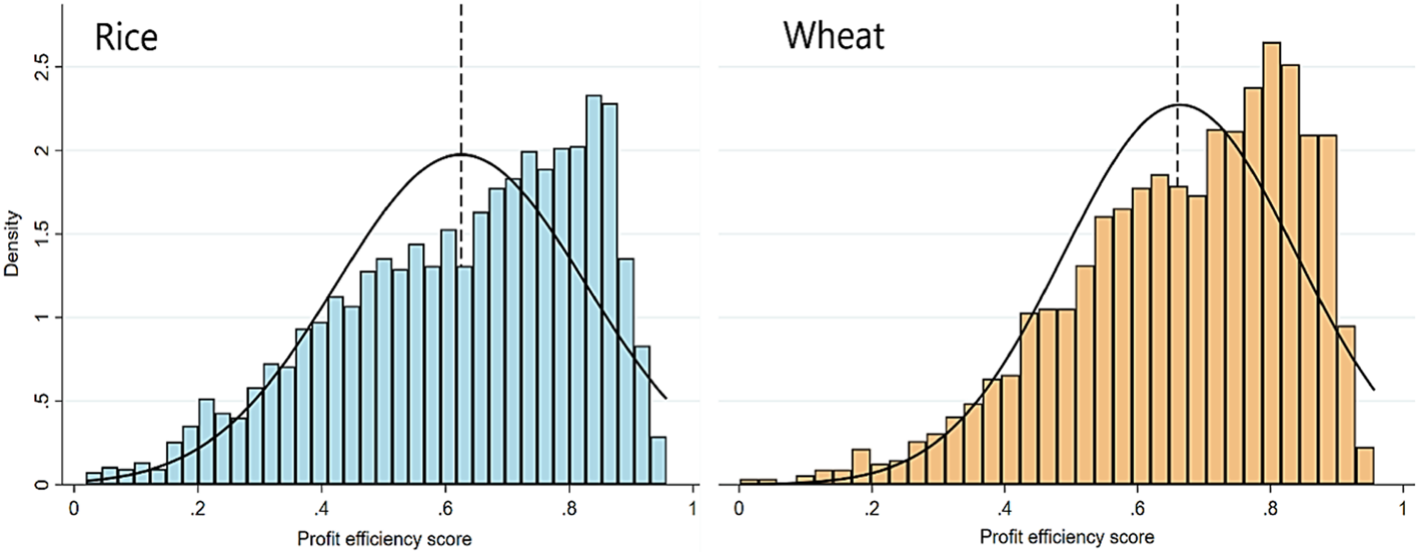
Distribution of profit efficiency in rice and wheat grown consecutively on the same field. Vertical dashed lines in both panels indicate the mean efficiency of rice (0.62) and wheat (0.65)

The mean profit efficiency scores (actual profit divided by potential profit) of rice and wheat fields were 0.62 and 0.65, respectively (Fig. 3). This implies that, on average, there is potential to raise profits by 38% in rice and 35% in wheat production at the current level of input use. We observed a similar distribution pattern of profit efficiency scores in both the crops, ranging widely from zero to above 0.9. These patterns were negatively skewed due to concentration of scores on the right side and the tail stretched longer toward the left. This construction was more striking in wheat fields. It highlighted the presence of large gaps among fields indicating a large inequality in profit efficiencies farmers are currently realizing. There are certain groups of farmers (toward right side of the means) who were able to apply inputs and deploy practices more reasonably, enabling them to achieve higher profits. Alternatively, there are many farmers in disadvantageous situations.

#### Difference by farm size class

**Fig. 4:**
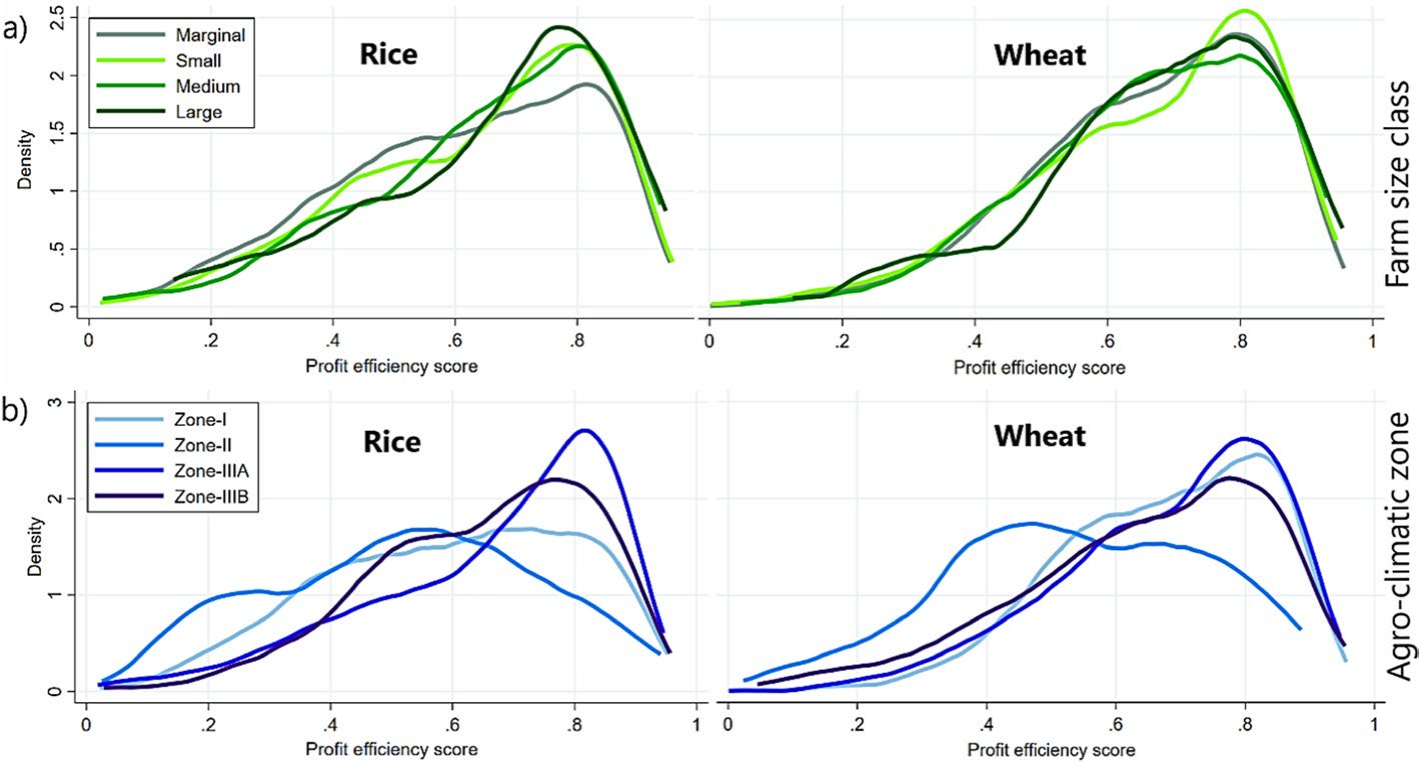
Comparison of profit efficiencies for rice and wheat: **a** Across different farm size categories, and **b** across various agro-climatic zones

For the rice fields, we observed minor differences in profit efficiency across the four farm size classes (Marginal: less than 1 ha, Small: between 1 and 2 ha, Medium: more than 2 to 4 ha, and Large: more than 4 ha). Marginal farmers recorded the lowest profit efficiency at 61%, as identified by the Kruskal–Wallis test (Fig. 4a). Profit efficiency then rose gradually with farm size, reaching 64% for small farmers to eventually 66% for large farmers. Despite this progression, the overall variation in profit efficiency in rice production was trivial. The wheat fields exhibited virtually no differences across farm size classes. The only exception was a marginal increase of 1% for large farmers compared to other classes.

#### Difference by agro-climatic zone

The differences in profit efficiency across geographically separated agro-climatic zones were more pronounced in both the crops. For rice, we found highest profit efficiency in Zone-IIIA (66%), followed closely by Zone-IIIB (65%) (Fig. 4b). Together, these two southern zones of Bihar appear to be relatively more profit efficient in rice production compared to other parts of the state. For wheat, the highest profit efficiencies were recorded in Zone-I and Zone-IIIA, both at 66%, highlighting Zone-IIIA as consistently the most efficient zone for both rice and wheat. In contrast, Zone-II, located in the northeastern part of Bihar, exhibited the lowest profit efficiencies, 52% for rice and 53% for wheat, respectively. This spatial disparity suggests that farmers in Zone-II may be constrained by unfavorable natural conditions or by external limitations, restricting their ability to generate profits at par with farmers in other zones.

### Spatial pattern in profit efficiency

We examined profit efficiency at a more granular geographical level, focusing on district-specific performance. The average profit efficiency for each district was calculated for both rice and wheat and subsequently mapped to visualize the relative standing of districts and the spatial differences across the state. To facilitate interpretation, profit efficiencies were categorized into three levels based on the observed state-wide range: low (50–60%), moderate (60–70%), and high (70–80%). For rice, 9 out of the 12 covered districts in north Bihar fell within the low efficiency category. In contrast, most districts in the south displayed moderate efficiency, with three districts achieving high efficiency. For wheat, almost all districts fell into either the moderate or high efficiency categories, with the exception of districts in Zone-II, which recorded comparatively lower profit efficiency. Crop-wise profit efficiency maps of districts are presented in Appendix 3.

**Fig. 5:**
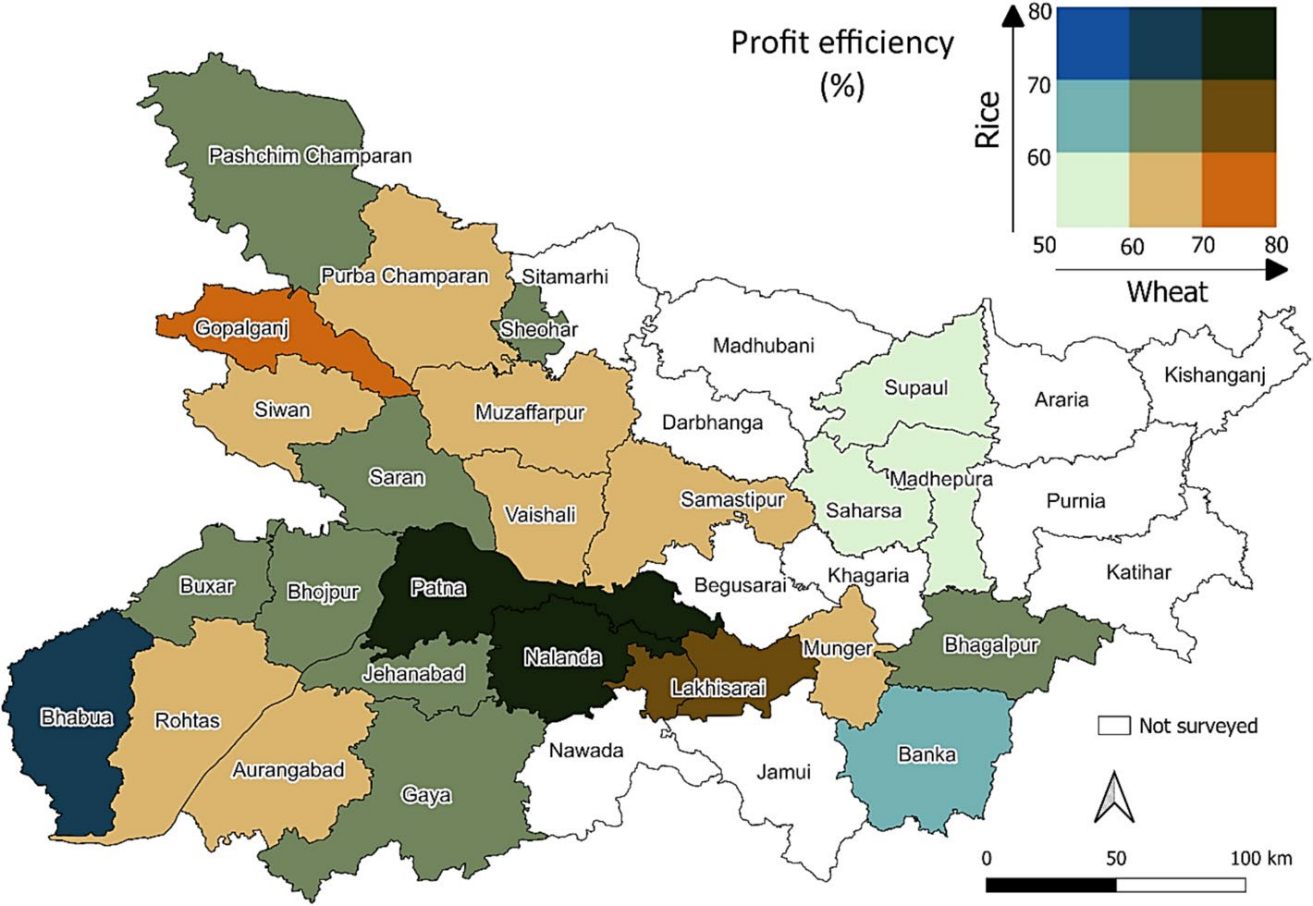
Combined profit efficiency map of rice and wheat across districts of Bihar

To further understand the combined performance of both crops, district-specific profit efficiencies for rice and wheat were combined in a bivariate choropleth map (Fig. 5). This presented districts with strong and weak performances in both the crops simultaneously. Patna, the state’s capital, along with nearby Nalanda district, exhibited higher profit efficiency in both rice and wheat production. Generally, districts in western part of Bihar showed moderate profitability for both crops. Beyond the capital region, the highest efficiency for rice was observed in Bhabua (southwest), while Gopalganj (northwest) recorded the highest efficiency for wheat.

The Moran’s I test results suggested some degree of spatial clustering in efficiency outcomes, with values of 0.204 for rice and 0.246 for wheat. These positive statistics indicate that neighboring fields may share certain unobserved characteristics. However, the relatively low magnitude of the coefficients implies that spatial dependence is weak, and efficiency differences are largely driven by already captured households, fields, soil, and weather variables. Outputs of the Moran’s I test for rice and wheat fields are available as Appendix 4.

### Factors affecting profit efficiency

**Fig. 6:**
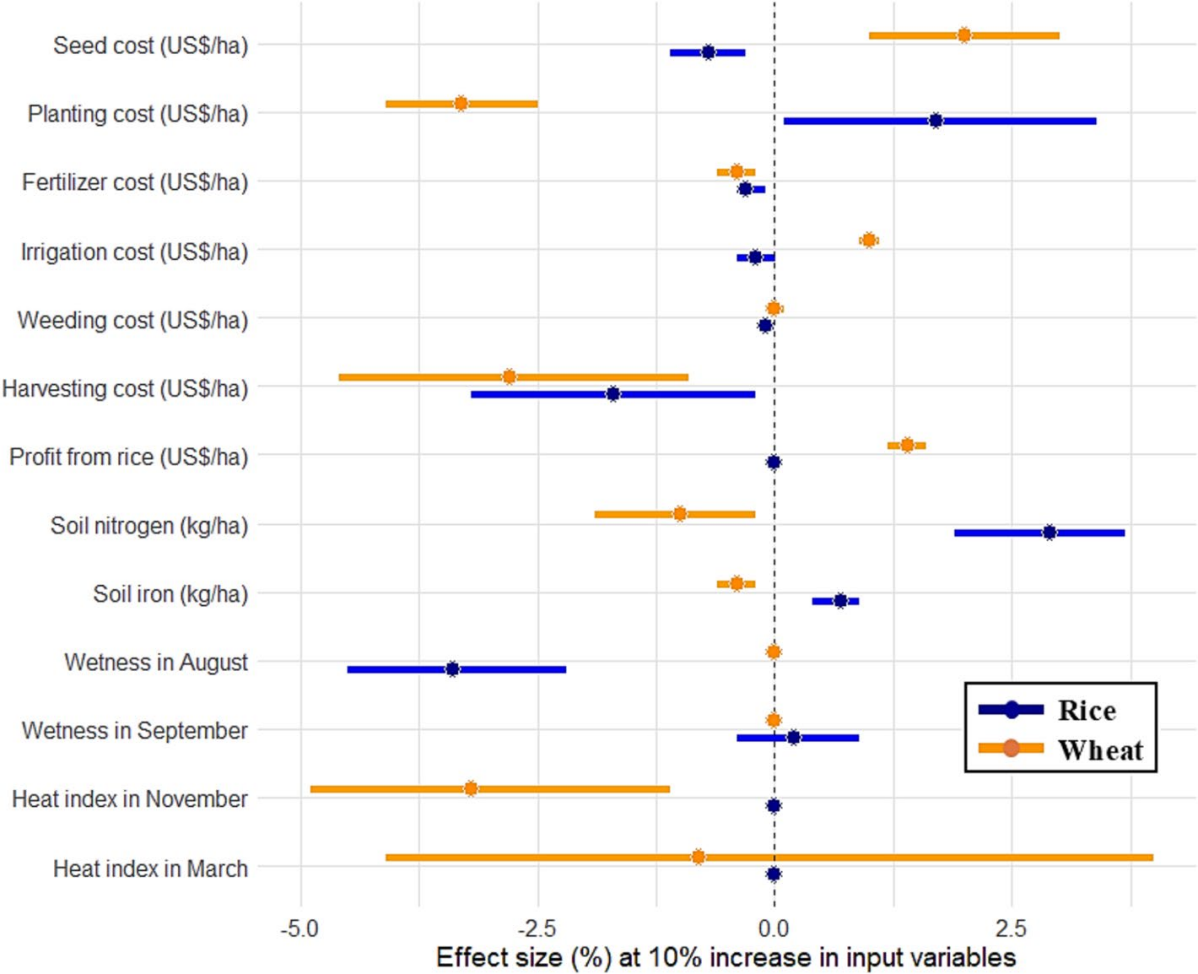
Parameter estimates for determinants of profit efficiency in rice and wheat fields (Dependent variable = Profit in US$ per hectare)

Based on the estimates of the profit frontier model, we computed effect size for a 10% increase in variable inputs. We found that profit from rice falls short to its potential primarily due to expensive harvesting operations, unfavorable rainfall and costly seed to a lesser extent. The concentration of rainfall in August, implying that previous months remain relatively drier, was a powerful factor in lowering profits. A 10% increase in the cost of rice harvesting (including threshing of grain) reduces the profit by nearly 1.8% (Fig. 6). We detected depletion in profit of 3.5% if rainfall in August exceeds the normal by 10%. While the directional effect of irrigation, fertilizers, and weed management costs were also negative on profit, these effects were unsubstantial.

Two cost variables with fairly high effect size in wheat were planting and harvesting operations. An increase of 10% in these two cost factors reduces profit from wheat production by 3.4% and 2.9%, respectively. We found that a warmer temperature in November is detrimental to profit from wheat, with an effect size of nearly −3.2%. Interestingly, we noticed that profit earned in rice positively affects profit in the immediate next wheat crop, with an effect size of 1.4%. In general, cost-related factors with positive effect size indicate that the investment in these inputs had been sub-optimal, and farmers could potentially invest more without negatively affecting their profits.

The model diagnostic computed the Gamma-value ($\gamma$) for rice and wheat, which were 0.946 and 0.916, respectively. This suggests that the variations in profit efficiencies were mainly due to inefficiencies and little (0.5% in wheat and 0.8% in rice) can be attributed to statistical noise in the data. To check the robustness of the frontier, we cross-validated these results from the trans–log specification with the semi–log and linear (no–log) functional forms. The results across these specifications were qualitatively consistent, indicating that the main findings regarding profit efficiency and the relative role of explanatory variables were not sensitive to the choice of functional form. We present the comparative table of three functional forms in Appendix 5. With cluster-robust standard errors analysis, we recognized that neighboring fields share common contextual factors that cannot be directly observed but are likely to generate dependence in the error structure.

### Sources of profit inefficiency

**Fig. 7:**
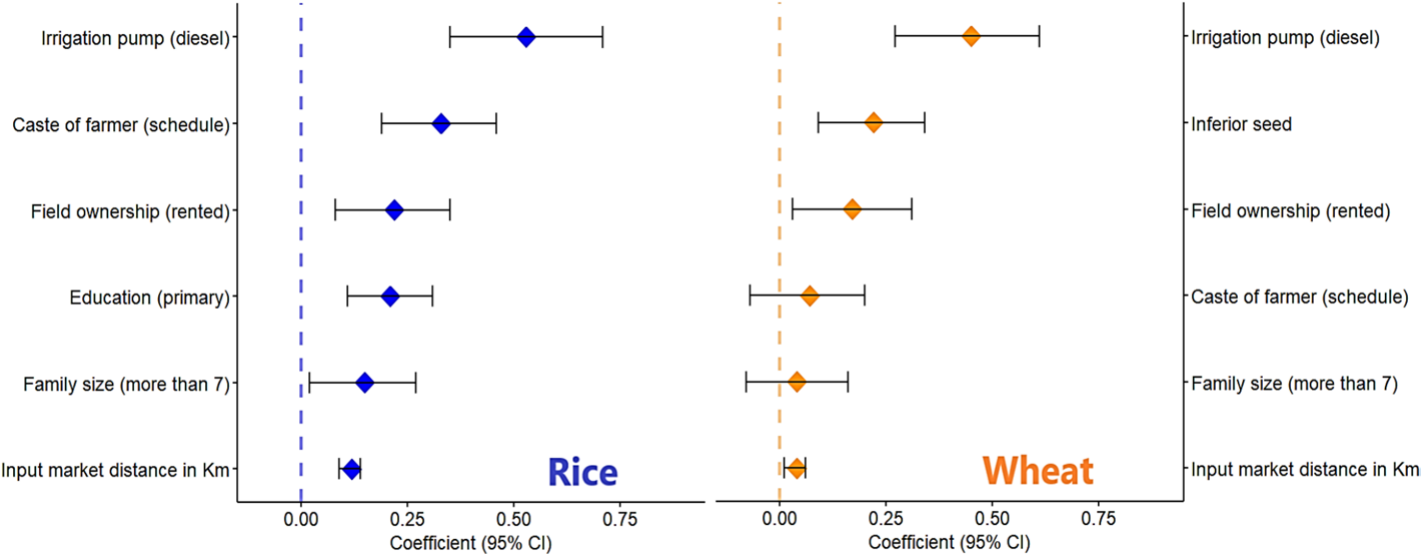
Major sources of profit inefficiency in the rice–wheat cropping system

The use of diesel pumps for irrigation was the most powerful source of inefficiency in both rice and wheat fields (Fig. 7). Rice fields irrigated with diesel pumps were merely half as efficient as those with electricity-driven pumps. The situation was slightly less severe for wheat fields—farmers using diesel pumps for irrigating wheat were found to make 45% less profit. Household characteristics, such as caste, field ownership type, and family size, were important sources of inefficiency. These were more commanding in rice production than wheat. The education level of farmers appeared to be another driver of inefficiency in rice cultivation. Aggregately, farmers educated only up to class five (primary level) made 21% less profit in rice than farmers having higher educational status. Distance of farm input markets negatively affected efficiency of rice production—one kilometer further adds 12% additional inefficiency. This connection was less noticeable in wheat. We detected that the selection of high-yielding seed variety was decisive in wheat production. Farmers using inferior wheat seeds were 22% less profit efficient.

## Discussion

We estimated profit efficiency and its determinants, including sources of inefficiency, for the two most important crops in Bihar, India. Drawing on data from more than 4000 farmers’ fields, our profit frontier model integrated costs, prices, socioeconomic, and field-specific variables such as weather and soil. The results demonstrate that in-season weather variability, weak irrigation infrastructure, and to a lesser extent, household characteristics are key drivers of profitability in rice and wheat. We also uncovered pronounced spatial heterogeneity in profit efficiency, pointing to the need for spatially differentiated policy interventions.

The computation of profit efficiency revealed the untapped profit potential that farmers could realize without additional input and costs. Our results highlight that farmers in Bihar could increase their profits by 38% in rice and 35% in wheat if inefficiencies were tackled. While several studies have measured profit efficiency, these were focused on a single crop and relied on smaller samples. Comparative evidence from other regions shows that profit gaps (potential–actual) in rice amount to 23% in Bangladesh (Rahman 2003), 19% in Brunei (Galawat and Yabe 2012), and 24% in Tanzania (Anna and Damas 2024), driven by technical, allocative, or scale inefficiencies. Although frontier estimates are not strictly comparable across regions, the profit augmentation potential in our study area is notably higher, highlighting the severity of resources being lost in Bihar.

In wheat production, profit efficiency was slightly better compared to rice production and almost matched with several other regions of global South. The potential profit gains reported elsewhere amount to 38% in Iraq (Mahmood et al. 2018), 37% in Ethiopia (Asfaw et al. 2019), and 27% in Pakistan (Mahmood et al. 2020). More importantly, the nature of inefficiency is highly context-specific, as the underlying determinants vary across regions. In Bihar, addressing inefficiency requires a tailored set of interventions, particularly the promotion of mechanization, upgrading of irrigation infrastructure, and adoption of weather-resilient production practices.

### Mechanization as a catalyst for profitability

Our study underscored the substantial potential of mechanization to enhance profitability in Bihar’s rice–wheat production system. Small landholdings and fragmented fields result in predominantly manual operations, with mechanization largely limited to land preparation and threshing in wheat (Shambhu and Jha 2012). Harvesting is particularly labor intensive and costly, exacerbated by rural outmigration, which disproportionately reduces profits, especially in wheat due to its narrow harvesting window (Das et al. 2020). Wheat planting also incurs high costs due to multiple rounds of conventional tillage led by intensive tractor use.

Mechanized interventions could markedly reduce these cost pressures. Zero-tillage wheat planting improves productivity and efficiency while lowering tillage-related costs (Krishna and Veettil 2014; Erenstein and Laxmi 2008). Yet, its adoption in Bihar remains limited due to knowledge and access gap (Keil et al. 2017). Similarly, combine harvester use is confined to only 15% of mostly large-scale farmers (Urban Cordeiro et al. 2024), leaving smallholders dependent on expensive manual labor. Our findings indicate that mechanization of harvesting and wheat planting could substantially improve profitability for smallholders. Collectively, these interventions could enhance the overall profit efficiency in both rice and wheat production in Bihar, mitigating labor constraints.

### Affordable irrigation solutions

Our results highlighted that costly diesel-based irrigation is a major source of inefficiency in Bihar’s rice–wheat production system. While rice is largely rainfed, wheat remains under irrigated due to the high expense of diesel-powered pumps, which remain the dominant source of irrigation energy in most areas of the state (Srivastava et al. 2021). Diesel costs are approximately four times higher than electric alternatives and although state subsidies partially offset these costs, they do not fully resolve the challenging economic conditions (Beniwal and Kishore 2024). Limited canal irrigation exists in some districts of Zone-IIIA, but it is often unreliable and insufficient. The recent expansion of rural electricity and promotion of electric pumps through state programs provides farmers with a viable, lower-cost alternative. Our findings indicate that affordable and reliable irrigation can substantially reduce profit inefficiencies and increase profitability. Therefore, investment in cost-effective and equitable irrigation infrastructure emerges as a critical lever for enhancing profit efficiency and addressing rural poverty in Bihar.

### Weather variability and profitability

The underdeveloped irrigation system in Bihar forces farmers to rely heavily on monsoon rainfall for rice establishment and subsequent water requirements for the rice crop. While normal monsoon pattern from June to September generally align with the rice-growing season and support production, increasing climate variability is undermining this reliability. Over the past three decades, Bihar has experienced high intra-annual rainfall variability and more extreme events (Zakwan and Ara 2019; Tesfaye et al. 2017). Our findings showed that excessive rainfall in August negatively impacts rice profitability. On the other hand, post-monsoon rains coincide with harvests, deteriorating grain quality and lowering market prices (Dixit and Pandey 2024).

Winter temperature trends are also shifting unfavorably and affecting wheat production. Cool nights, critical for wheat growth and development have become less frequent (Subash et al. 2013). Additionally, minimum temperatures during winter have increased (Rao et al. 2015). Our analysis established that higher November temperatures above long-term means reduce profit in wheat production. These results underscored the urgent need to adapt current production practices to emerging climatic stresses. Development and deployment of climate-resilient rice and wheat cultivars, coupled with adaptive agronomic practices, are essential strategies to safeguard yields and profitability under changing weather patterns.

### Natural constraint in Zone-II

Spatial mapping of profit efficiencies revealed that Zone-II in northeastern Bihar exhibits consistently lower profitability for both rice and wheat. This pattern is closely linked to natural challenges associated with the region’s geography. Apart from the Ganges, multiple rivers such as Bagmati, Gandak, Kamla, Koshi, and Mahananda enter this region from the Himalaya, causing frequent flooding, particularly during September and October (Jha and Gundimeda 2019). As a low-lying plain, floodwater often stagnates, submerging rice fields and delaying wheat planting in this zone. Recent studies indicate that both the magnitude and duration of flooding have increased, heightening vulnerability in the zone (Sahani et al. 2023). These findings suggest that addressing flood risks is a prerequisite for any agricultural development interventions to succeed including profitability enhancement measures.

Limitations and future research: The value of crop residues couldn’t be captured due to unorganized markets and uncertain pricing. Rice residue is often burnt or left in fields, with collected portions rarely sold in measurable ways. Wheat residue is mainly used as cattle feed, with occasional surplus sales at uncertain prices and times. Future studies should explore mechanized harvesting solutions and develop business models that are practical and sustainable for smallholders. Similar attention is needed for mechanized wheat planting. Irrigation costs should be evaluated by different types of users to identify the most suitable and efficient options. In addition, testing improved rice and wheat cultivars is important for strengthening farmers’ immediate adaptive capacity.

## Conclusion and policy implications

Our study examines how profits can be enhanced for more than 100 million smallholders in Bihar, India, who predominantly cultivate rice and wheat in rotation. Using stochastic profit frontier model applied to a large cross-sectional survey combined with geo-spatial data, we measured profit efficiency in the production of rice and wheat. We found scope to increase profits by about 38% in rice and 35% in wheat at current input levels. Labor-intensive harvesting was the main factor reducing profit efficiency, while high planting costs were particularly limiting profits in wheat. Weather shocks also played a role, with extreme rainfall during the mid-monsoon lowering rice profits and warmer early-winter temperatures affecting wheat. Inefficiencies were further linked to costly irrigation systems, especially dependence on deployment of diesel pumps. Spatial mapping highlighted specific areas where interventions could be prioritized.

Our findings suggest that smallholder profits could be raised substantially through mechanization, climate-adaptive practices, and cost-effective irrigation. Field-appropriate mechanized solutions for harvesting both crops and planting wheat can raise profitability. Transitioning to less-expensive alternatives for diesel-fueled irrigation system is one of the most important structural components. Coping with in-season weather variability remains complex, but accurate dissemination of weather forecasts and advisory services, alongside long-term investment in climate-resilient rice and wheat varieties, would strengthen farmers’ adaptive capacity. Taking together, these insights point out measures that could significantly improve profitability from rice and wheat production and contribute to economic well-being of smallholders.

## Data Availability

No datasets were generated or analysed during the current study.

## Appendix 1

Empirical single-step stochastic profit frontier models of rice

$${\text{ln}p}_{i}= {\beta }_{0}+ {\beta }_{1}\text{ln}{C}_{1i}+ {\beta }_{2}{\text{ln}C}_{2i}+ {\beta }_{3}{\text{ln}C}_{3i}+ {\beta }_{4}{\text{ln}C}_{4i}+ {\beta }_{5}{\text{ln}C}_{5i}+ {\beta }_{6}{\text{ln}C}_{6i}+ {\beta }_{7}{Z}_{1i}+ {\beta }_{8}{\text{ln}Z}_{2i}+ {\beta }_{9}\text{ln}{Z}_{3i}+ {\beta }_{10}\text{ln}{Z}_{4i}+ {\beta }_{11}\text{ln}{Z}_{5i}+ {V}_{i}- {U}_{i}$$

where, ${\text{ln}p}_{i}$ = profit from rice calculated as total revenue less variable cost of production, ${C}_{1}$ = average cost of field preparation and transplanting of rice in the field, ${C}_{2}$ = average cost of rice seeds used in the field, ${C}_{3}$ = average cost of all fertilizers applied in the field and cost of fertilizer application, ${C}_{4}$ = average cost incurred in applying total number of irrigations, ${C}_{5}$ = average cost of all manual weeding operations including herbicides and their application, ${C}_{6}$ = average cost of harvesting rice from the field and its threshing, ${Z}_{1}$ = day of transplanting rice nursery in the field, ${Z}_{2}$ = rainfall in August used as wetness index calculated based on long-term average, ${Z}_{3}$ = rainfall in September used as wetness index calculated based on long-term average, ${Z}_{4}$ = amount of nitrogen available in the soil, ${Z}_{5}$ = amount of iron available in the soil, ${\beta }_{0}- {\beta }_{11}$ = unidentified parameters to be measured, ${V}_{i}$ = random error assumed to be independently and identically distributed, and ${U}_{i}$ = inefficiency effects assumed to be non-negative and independently distributed.

We estimated inefficiency effects (${U}_{i}$) using profit inefficiency model specified in equation below:

$${U}_{i}= {\delta }_{0}+ {\delta }_{1}{X}_{1}+ {\delta }_{2}{X}_{2}+ {\delta }_{3}{X}_{3}+ {\delta }_{4}{X}_{4}+ {\delta }_{5}{X}_{5}+ {\delta }_{6}{X}_{6}+ {\delta }_{7}{X}_{7}+ {\delta }_{8}{X}_{8}$$

where, ${\delta }_{0}- {\delta }_{8}$ = unknown parameters to be estimated and ${X}_{1}$ is field size, ${X}_{2}$ is field ownership type, ${X}_{3}$ is irrigation pump type, ${X}_{4}$ is seed type, ${X}_{5}$ is distance of input market, ${X}_{6}$ is education of farmer, ${X}_{7}$ is family size, and ${X}_{8}$ is caste of farmer.

The single-step profit frontier model of wheat is similar to rice except that it has an additional parameter—*Z*_6_ as profit earned in previous rice.

$${\text{ln}p}_{i}= {\beta }_{0}+ {\beta }_{1}\text{ln}{C}_{1i}+ {\beta }_{2}{\text{ln}C}_{2i}+ {\beta }_{3}{\text{ln}C}_{3i}+ {\beta }_{4}{\text{ln}C}_{4i}+ {\beta }_{5}{\text{ln}C}_{5i}+ {\beta }_{6}{\text{ln}C}_{6i}+ {\beta }_{7}{Z}_{1i}+ {\beta }_{8}{\text{ln}Z}_{2i}+ {\beta }_{9}\text{ln}{Z}_{3i}+ {\beta }_{10}\text{ln}{Z}_{4i}+ {\beta }_{11}\text{ln}{Z}_{5i}+ {\beta }_{12}\text{ln}{Z}_{6i}+ {V}_{i}- {U}_{i}$$

where, ${\text{ln}p}_{i}$ = profit from wheat calculated as total revenue less variable cost of production, ${C}_{1}$ = average cost of field preparation and planting of wheat, ${C}_{2}$ = average cost of rice seeds used in the field, ${C}_{3}$ = average cost of all fertilizers applied in the field and cost of fertilizer application, ${C}_{4}$ = average cost incurred in applying total number of irrigations, ${C}_{5}$ = average cost of all manual weeding operations including herbicides and their application, ${C}_{6}$ = average cost of harvesting wheat from the field and its threshing, ${Z}_{1}$ = planting day of wheat, ${Z}_{2}$ = temperature of November used as heat index calculated based on long-term mean, ${Z}_{3}$ = temperature of March used as heat index calculated based on long-term mean, ${Z}_{4}$ = amount of nitrogen available in the soil, ${Z}_{5}$ = amount of iron available in the soil, ${Z}_{6}$ = average profit earned from previous rice crop, ${\beta }_{0}- {\beta }_{12}$ = unidentified parameters to be measured, ${V}_{i}$ = random error assumed to be independently and identically distributed, and ${U}_{i}$ = inefficiency effects assumed to be non-negative and independently distributed.

The inefficiency model to estimate ${U}_{i}$ in wheat production is exactly the same as of rice that has already been specified above.

## Appendix 2

**Table:** Descriptive statistics of variables used in profit efficiency measurement of rice

Variable	Description	Mean	Std. Dev
*Target variable*			
Profit	Revenue-production cost (US$ per hectare)	204.4	242.9
*Independent variables*			
Planting cost	Cost incurred in establishment of rice (US$ per hectare)	109.2	6.4
Seed cost	Cost of rice seed (US$ per hectare)	34.3	13.6
Fertilizer cost	Cost of fertilizers and their applications (US$ per hectare)	79.1	28.8
Irrigation cost	Cost incurred in applying irrigations (US$ per hectare)	112.4	73.3
Weeding cost	Cost of herbicides, spraying and weeding (US$ per hectare)	78.8	35.3
Pest control cost	Cost of fungi-/insecticides and spraying (US$ per hectare)	3.5	6.4
Harvesting cost	Cost incurred in harvesting and threshing (US$ per hectare)	149.3	10.2
Planting date	Transplanting day of rice (Julian day)	195.2	11.4
August wetness	(Actual monthly rainfall/long-term monthly normal rainfall) *100 in August	129.2	20.2
September wetness	(Actual monthly rainfall/long-term monthly normal rainfall) *100 in September	58.4	16.9
Soil nitrogen	Nitrogen content in the soil (kg per hectare)	268.5	44.8
Soil iron	Iron content in the soil (kg per hectare)	36.9	29.2
*Other control variables*			
Irrigation pump type	Dummy (=1 if diesel)	0.87	0.33
Field ownership type	Dummy (=1 if rented)	0.14	0.35
Rice seed variety	Dummy (=1 if other than hybrid)	0.67	0.47
Caste	Dummy (=1 if schedule)	0.15	0.35
Education	Dummy (=1 if up to class 5 i.e., primary)	0.31	0.46
Produce sold	Dummy (=1 if less than half of total produce)	0.88	0.32
Field size	Size of the field (hectare)	0.21	0.22
Farm size	Size of the total farm (hectare)	1.14	1.51
Family size	Number of household members	7.93	2.73
Market distance	Distance of the nearest input market (km)	4.36	4.09

**Table:** Descriptive statistics of variables used in profit efficiency measurement of wheat

Variable	Description	Mean	Std. Dev
*Target variable*			
Profit	Revenue-production cost (US$ per hectare)	176.9	188.6
*Independent variables*			
Planting cost	Cost incurred in establishment of rice (US$ per hectare)	105.3	14.9
Seed cost	Cost of rice seed (US$ per hectare)	31.8	4.9
Fertilizer cost	Cost of fertilizers and their applications (US$ per hectare)	69.6	35.3
Irrigation cost	Cost incurred in applying irrigations (US$ per hectare)	45.3	17.5
Weeding cost	Cost of herbicides, spraying, and weeding (US$ per hectare)	8.6	9.1
Harvesting cost	Cost incurred in harvesting and threshing (US$ per hectare)	121.5	11.3
Planting date	Sowing day of wheat (Julian day)	337.4	12.2
November heat index	(Actual monthly mean temperature/long-term monthly mean temperature) *100 in November	90.8	2.0
March heat index	(Actual monthly mean temperature/long-term monthly mean temperature) *100 in March	96.8	1.9
Soil nitrogen	Nitrogen content in the soil (kg per hectare)	268.5	44.8
Soil iron	Iron content in the soil (kg per hectare)	36.9	29.2
Profit in previous rice crop	Profit earned from the previous rice crop (US$ per hectare)	204.4	242.9
*Other control variables*			
Irrigation pump type	Dummy (=1 if diesel)	0.87	0.33
Field ownership type	Dummy (=1 if rented)	0.14	0.35
Wheat seed type	Dummy (=1 if inferior)	0.58	0.49
Caste	Dummy (=1 if schedule)	0.15	0.35
Education	Dummy (=1 if up to class 5 i.e., primary)	0.31	0.46
Produce sold	Dummy (=1 if less than half of total produce)	0.88	0.32
Field size	Size of the field (hectare)	0.21	0.22
Farm size	Size of the total farm (hectare)	1.14	1.51
Family size	Number of household members	7.93	2.73
Market distance	Distance of the nearest input market (km)	4.36	4.09

## Appendix 3

Profit efficiency of rice production in the districts of Bihar.

Profit efficiency of wheat production in the districts of Bihar.

## Appendix 4

- Moran’s I test results for spatial autocorrelation in rice fields.
- Moran’s I test results for spatial autocorrelation in wheat fields.

## Appendix 5

**Table:** Comparison of parameter estimates using Log–Log, Semi–Log, and No–Log models (*N* = 4016)

	Log–Log model				Semi–Log model				No–Log model			
	Coefficient		Std. Err		Coefficient		Std. Err		Coefficient		Std. Err	
*Variables for profit in rice*												
Seed cost	−0.059		0.020		−0.001		0.001		−1.928		0.260	
Planting cost	0.185		0.082		0.009		0.001		3.755		0.629	
Fertilizer cost	−0.025		0.010		−0.001		0.000		−0.343		0.116	
Irrigation cost	−0.013		0.012		−0.001		0.000		−0.294		0.051	
Weeding cost	−0.010		0.003		−0.002		0.000		−0.544		0.097	
Harvest cost	−0.176		0.074		0.001		0.001		−1.108		0.351	
Soil nitrogen	0.283		0.046		0.003		0.000		1.175		0.100	
Soil iron	0.073		0.011		0.001		0.000		0.452		0.128	
Wetness in August	−0.346		0.061		−0.008		0.001		−3.004		0.285	
Wetness in September	0.024		0.034		0.001		0.001		0.348		0.352	
Constant	9.196		0.694		6.782		0.267		718.608		114.943	
Log likelihood	−2254.9				−2821.9				−2.71E + 04			
Wald *χ*^2^ (14)	747.6				1018.3				1242.3			
*Variables for profit in wheat*												
Seed cost		0.240		0.052		0.007		0.002		8.718		0.570
Planting cost		−0.340		0.040		−0.005		0.000		−2.386		0.170
Fertilizer cost		−0.041		0.012		−0.002		0.000		−0.591		0.073
Irrigation cost		0.103		0.006		0.005		0.000		1.915		0.140
Weeding cost		0.005		0.001		0.003		0.001		1.294		0.281
Harvest cost		−0.294		0.098		−0.003		0.001		−0.844		0.229
Profit from rice		0.144		0.011		0.001		0.000		0.183		0.011
Soil nitrogen		−0.091		0.042		−0.001		0.000		−0.222		0.068
Soil iron		−0.03		0.012		−0.001		0.000		−0.207		0.097
Heat index in November		−0.321		0.275		−0.024		0.005		-9.433		1.689
Heat index in March		−0.080		0.378		−0.008		0.005		–−1.005		1.567
Constant		4.944		2.680		7.539		0.787		10.039		263.646
Log likelihood		−2115.8				−2455.2				−2.58E + 04		
Wald *χ*^2^ (14)		1315.8				1579.3				2087.8		
